# Supplementary material for: Immunopeptidomics of cutaneous leishmaniasis patients reveals the natural antigenic landscape
Source: Front Immunol. 2026 Feb 20;17:1765843. doi: 10.3389/fimmu.2026.1765843 (PMC12963358; doi:10.3389/fimmu.2026.1765843)
Supplement: Supplementary file 1 [file DataSheet1.docx]

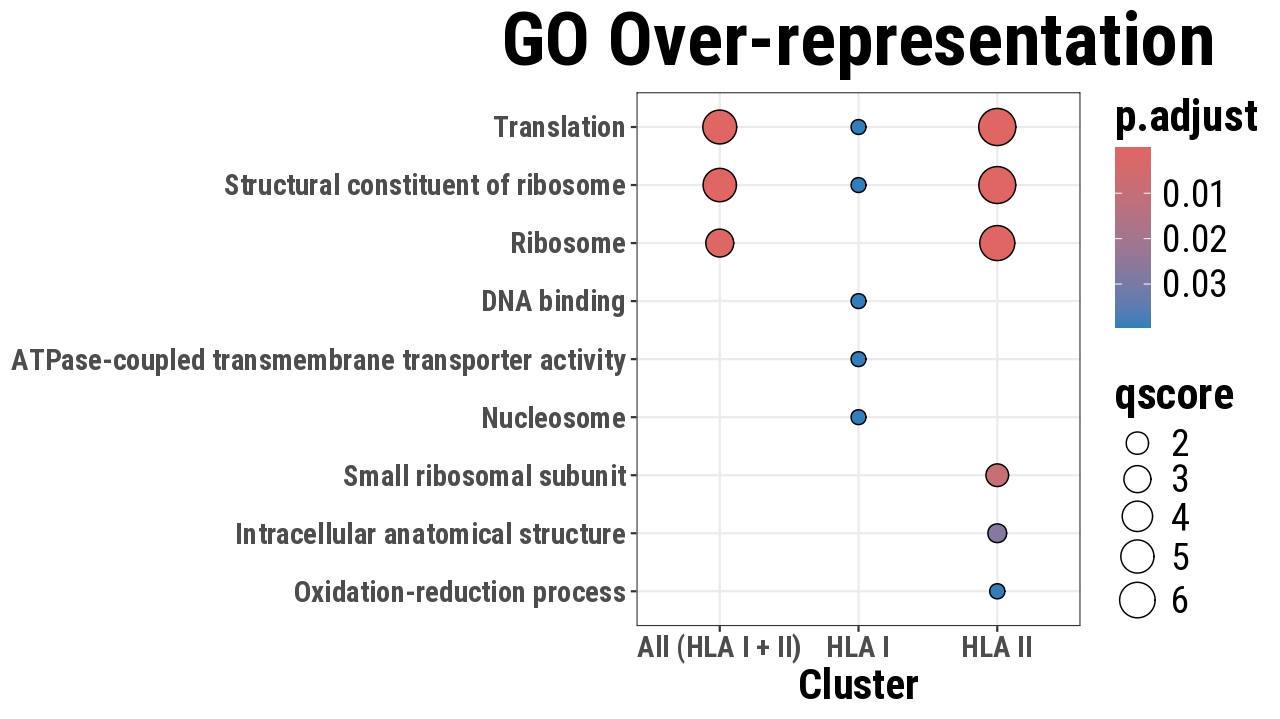


**Supplementary Figure 4**. GO over-representation test on all MHC-presented Leishmania antigens. P-values were adjusted by the Benjamini-Hochberg method.
