# Supplementary material for: Immunopeptidomics of cutaneous leishmaniasis patients reveals the natural antigenic landscape
Source: Front Immunol. 2026 Feb 20;17:1765843. doi: 10.3389/fimmu.2026.1765843 (PMC12963358; doi:10.3389/fimmu.2026.1765843)
Supplement: Supplementary file 2 [file DataSheet2.docx]

**
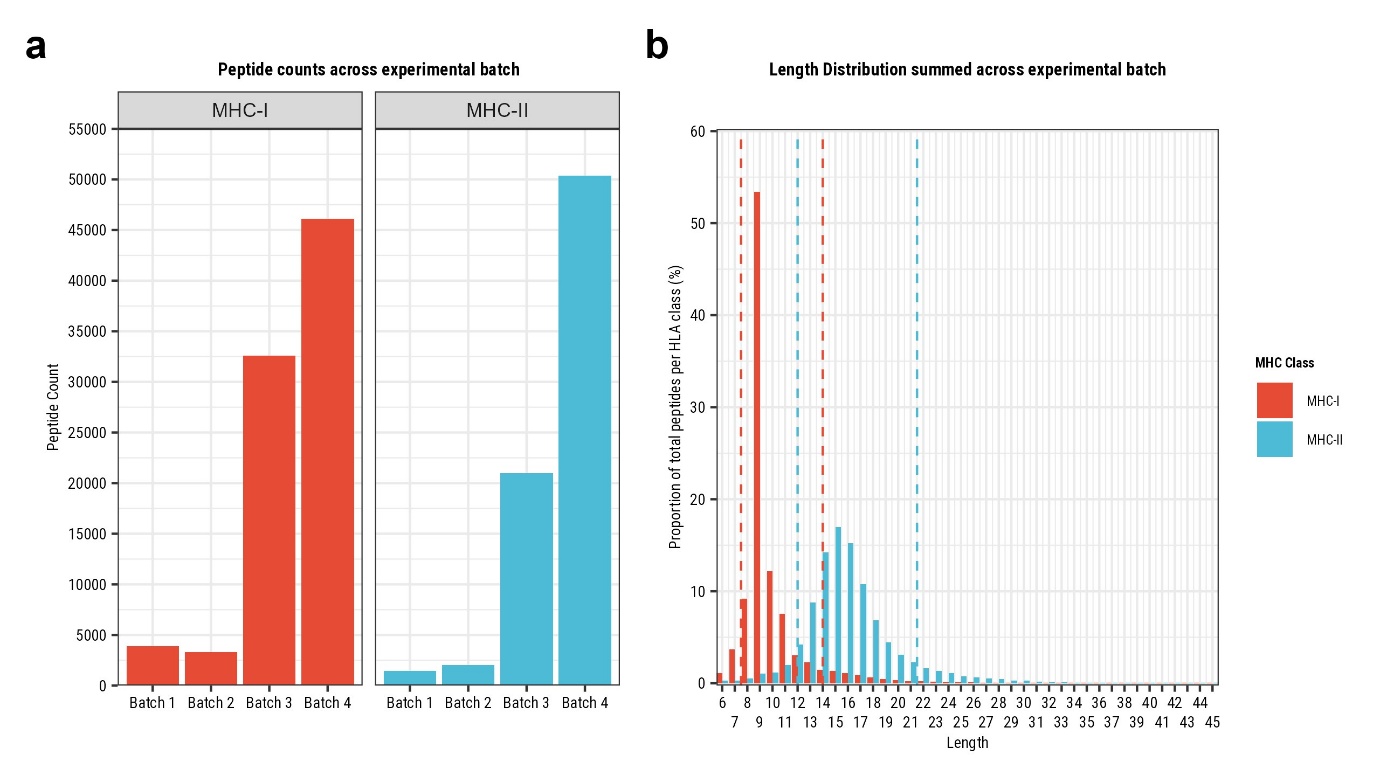
**

**Supplementary Figure 1**. MHC-presented peptide counts and the lengths of these peptides across experiments. (A) The peptide count per experiment by HLA class, in red for HLA-I and blue for HLA-II. (B) The length distribution of all MHC-presented peptides by HLA class, in red for HLA-I and blue for HLA-II. The red dashed lines represent the 8-12 length threshold for selecting higher-confidence MHC-I peptides, and the blue dashed lines represent the 12-21 length threshold for selecting higher-confidence MHC-II peptides.
