# Supplementary material for: Immunopeptidomics of cutaneous leishmaniasis patients reveals the natural antigenic landscape
Source: Front Immunol. 2026 Feb 20;17:1765843. doi: 10.3389/fimmu.2026.1765843 (PMC12963358; doi:10.3389/fimmu.2026.1765843)
Supplement: Supplementary file 3 [file DataSheet3.docx]

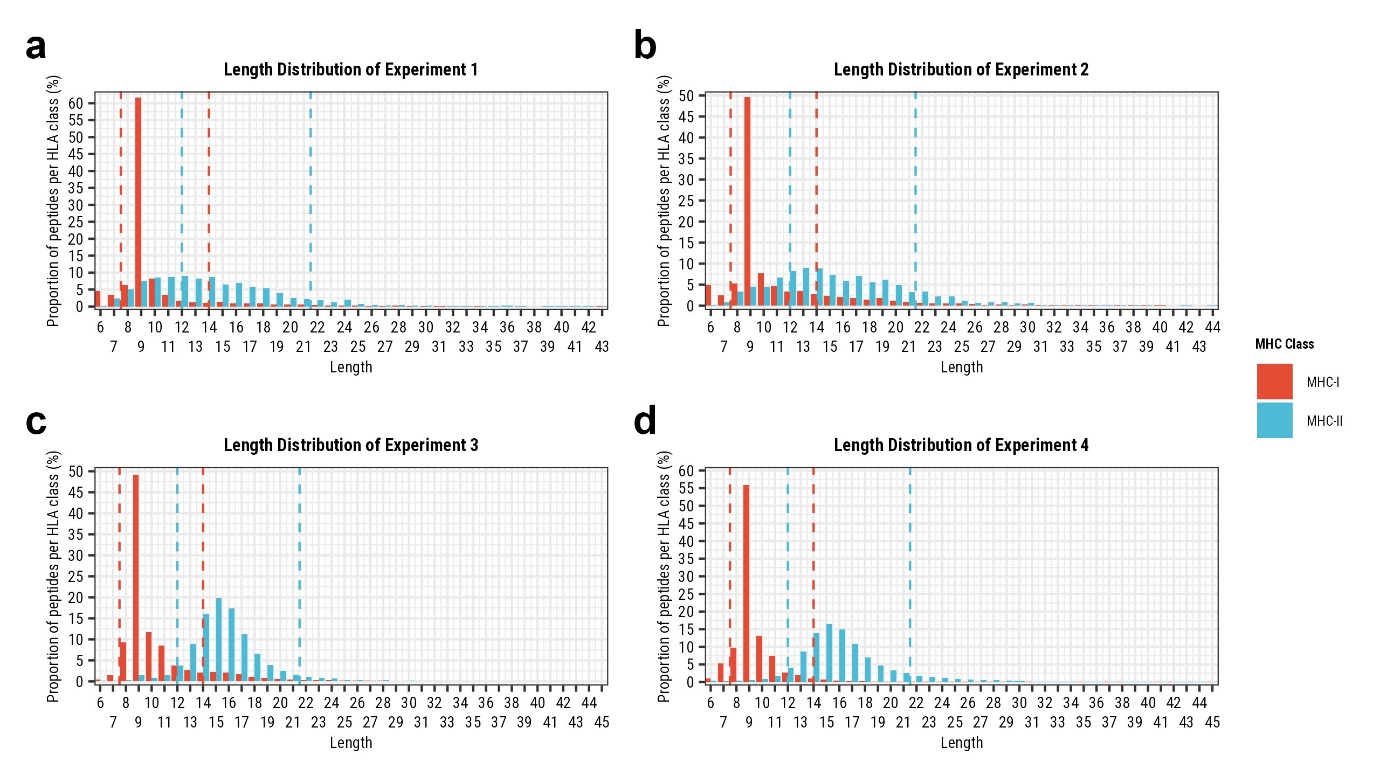


**Supplementary Figure 2**. The lengths of the MHC-presented peptides identified across experiments for A) experimental batch 1, B) experimental batch 2, C) experimental batch 3, and D) experimental batch 4. The red dashed lines represent the 8-12 length threshold for selecting higher-confidence MHC-I peptides, and the blue dashed lines represent the 12-21 length threshold for selecting higher-confidence MHC-II peptides.
